# The Calcium-Dependent Protein Kinase CPK33 Mediates Strigolactone-Induced Stomatal Closure in *Arabidopsis thaliana*

**DOI:** 10.3389/fpls.2019.01630

**Published:** 2019-12-17

**Authors:** Xuening Wang, Shuo Lv, Xiangyu Han, Xiongjuan Guan, Xiong Shi, Jingke Kang, Luosha Zhang, Bing Cao, Chen Li, Wei Zhang, Guodong Wang, Yonghong Zhang

**Affiliations:** ^1^ National Engineering Laboratory for Resource Development of Endangered Crude Drugs in Northwest China, Key Laboratory of Medicinal Resources and Natural Pharmaceutical Chemistry, Ministry of Education, College of Life Sciences, Shaanxi Normal University, Xi’an, China; ^2^ Laboratory of Medicinal Plant, Institute of Basic Medical Sciences, School of Basic Medicine, Biomedical Research Institute, Hubei Key Laboratory of Wudang Local Chinese Medicine Research, Hubei University of Medicine, Shiyan, China; ^3^ Key Laboratory of Plant Development and Environment Adapting Biology, Ministry of Education, School of Life Science, Shandong University, Qingdao, China; ^4^ Hubei Key Laboratory of Embryonic Stem Cell Research, Hubei University of Medicine, Shiyan, China

**Keywords:** Ca^2+^, calcium-dependent protein kinase, guard cell, stomatal closure, strigolactones

## Abstract

Strigolactones (SLs) are known to mediate plant acclimation to environmental stress. We recently reported that SLs acted as prominent regulators in promotion of stomatal closure. However, the detailed mechanism by which SLs induce stomatal closure requires further investigation. Here we studied the essential role of the calcium (Ca^2+^) signal mediating by the calcium-dependent protein kinase (CPK) in SL-induced stomatal closure. SL-induced stomatal closure was strongly inhibited by a Ca^2+^ chelator and Ca^2+^ channel blockers, indicating that Ca^2+^ functions in SL promotion of stomatal closure. Through examining a collection of *cpk* mutants, we identified CPK33, potentially acting as a Ca^2+^ transducer, which is implicated in guard cell SL signaling. SL- and Ca^2+^-induced stomatal closure were impaired in *cpk33* mutants. CPK33 kinase activity is essential for SL induction of stomatal closure as SL-induced stomatal closure is blocked in the dead kinase mutant of CPK33. The *cpk33* mutant is impaired in H_2_O_2_-induced stomatal closure, but not in SL-mediated H_2_O_2_ production. Our study thus uncovers an important player CPK33 which functions as an essential Ca^2+^ signals mediator in *Arabidopsis* guard cell SL signaling.

## Introduction

Plants are sessile organisms that confront with a wide range of biotic and abiotic stress conditions during their life cycle. As a strategy to cope with environmental stress, plants utilize stomatal pores, each consisting of a pair of guard cells, that open and close to modulate gas exchange for photosynthesis, transpirational water loss, and stomatal immunity, thereby allowing plants to respond properly to diverse environmental stress (Hetherington and Woodward, 2003; Ruszala et al., 2011; Blatt et al., 2017; Melotto et al., 2017). Guard cells have developed sophisticated mechanisms which enable plants to appropriately control of stomatal apertures in mediating response of environmental stimuli such as light, drought, and external calcium ion (Ca^2+^) (Kim et al., 2010; Murata et al., 2015). Particularly, phytohormones, including abscisic acid (ABA), ethylene, brassinosteroids, strigolactones (SLs), salicylic acid (SA), jasmonic acid (JA), and small signaling peptides have been found to play pivotal roles through their coordination with various key guard cell signaling components to modulate stomatal apertures in response to fluctuating environmental stress (Kim et al., 2010; Daszkowska-Golec and Szarejko, 2013; Munemasa et al., 2015; Murata et al., 2015; Cardinale et al., 2018; Mostofa et al., 2018; Qu et al., 2019; Zhang et al., 2019).

Other than their notable roles in shoot branching (Al-Babili and Bouwmeester, 2015), SLs have been found to be implicated in many plant developmental processes such as primary root development (Kapulnik et al., 2011; Ruyter-Spira et al., 2011), adventitious root formation (Rasmussen et al., 2012; Sun et al., 2015), secondary growth (Agusti et al., 2011), photomorphogenesis (Shen et al., 2007; Shen et al., 2012), flower development (Snowden et al., 2005; Kohlen et al., 2012; Liu et al., 2013), and hypocotyl elongation (Tsuchiya et al., 2010; Jia et al., 2014). Notably, accumulating data indicated that SLs are also involved in mediating plant responses to environmental stress, rendering plants to defend against abiotic stress as well as against specific bacterial and fungal species (Marzec, 2016; Cardinale et al., 2018; Mostofa et al., 2018). Specifically, SL-deficient and SL-signaling mutants exhibited drought hypersensitivity, whereas SLs application rescued drought-sensitive phenotypes of SL-deficient mutants and strengthened drought tolerance of wild-type (WT) plants (Bu et al., 2014; Ha et al., 2014; Liu et al., 2015; Li et al., 2017; Zhang et al., 2018). We recently revealed that SLs could induce stomatal closure through enhancing hydrogen peroxide (H_2_O_2_) and nitric oxide production in an ABA-independent manner, possibly preventing water loss and pathogen invasion and thereby resulting in plant acclimation to environmental stress (Lv et al., 2018; Zhang et al., 2018). However, the detail molecular mechanism, especially the intracellular events that are initiated by SLs in guard cells, remains largely unclear. To this end, the potential downstream component(s) that transduces guard cell SL signaling is thus required to be determined.

It has long been known that calcium functions as a secondary messenger in stomatal closure (Blatt, 2000; Bowler and Fluhr, 2000; Kim et al., 2010; Murata et al., 2015; Ray, 2017). For instance, through H_2_O_2_ activation of Ca^2+^-permeable cation channels, ABA triggers an increment of cytosolic Ca^2+^ [(Ca^2+^)_cyt_] that includes Ca^2+^ influx elevation from extracellular spaces and Ca^2+^ release from intracellular stores (Pei et al., 2000). In addition, JA-induced stomatal closure is mediated by cytosolic Ca^2+^ since JA signaling in guard cells is inhibited by Ca^2+^ channel blockers (Suhita et al., 2003; Suhita et al., 2004). Likewise, Ca^2+^ signaling is found to be implicated in SA induction of stomatal closure in a mode of action similar to studies of ABA- and JA-mediated stomatal closure (Prodhan et al., 2018). Eventually, the resultant guard cell cytosolic Ca^2+^ elevation promotes stomatal closure by stimulation of SLOW ANION CHANNEL-ASSOCIATED 1 (SLAC1) anion channels and/or the GATED OUTWARDLY-RECTIFYING K^+^ (GORK) channel (Kim et al., 2010; Murata et al., 2015; Roux and Leonhardt, 2018).

Calcium-dependent protein kinases (CPKs) function as Ca^2+^ signal transducers involving in various biological processes including Ca^2+^-dependent guard cell signaling (Boudsocq and Sheen, 2013; Schulz et al., 2013; Simeunovic et al., 2016). Mechanically, CPKs are known to activate SLAC1 and GORK channels to induce stomatal closure (Kim et al., 2010; Boudsocq and Sheen, 2013; Schulz et al., 2013; Simeunovic et al., 2016; Corratgé-Faillie et al., 2017). To date, a number of CPKs have been identified to be implicated in guard cell signaling to mediate stomatal movement. Several CPKs, including CPK3, CPK4, CPK6, CPK8, CPK9, CPK10, CPK11, and CPK33, are involved in ABA-mediated stomatal closure through distinct modes of action (Boudsocq and Sheen, 2013; Schulz et al., 2013; Li C.L et al., 2016; Chen et al., 2019). For instance, CPK3 and CPK6 activated ABA-induced stomatal closure and slow-type (S-type) anion channel activity (Mori et al., 2006). Disruption of *CPK6* impaired JA-mediated stomatal closure and S-type anion channels activation, implying that CPK6 acted as a positive regulator in guard cell MeJA signaling (Munemasa et al., 2011). CPK3 and CPK6 functioned additively in SA-induced stomatal closure and SA activation of S-type anion channels (Prodhan et al., 2018). CPK33 suppressed ABA-induced stomatal closure and S-type anion channel activity (Li C.L et al., 2016; Chen et al., 2019), whereas CPK33 stimulated GORK activity to promote stomatal closure (Corratgé-Faillie et al., 2017). It was reported that CPK10, possibly association with HEAT SHOCK PROTEIN 20-LIKE PROTEIN 1 (HSP1), functioned in ABA- and Ca^2+^-mediated stomatal closure in response to drought stress (Zou et al., 2010). Altogether, these findings underscore the importance of CPKs in the modulation of stomatal closure. CPKs and Ca^2+^-independent kinases [e.g. SnRK2-type protein kinase OPEN STOMATA1 (OST1)] have long been recognized to be involved in activating ion channels and stimulating stomatal closure (Geiger et al., 2009; Lee et al., 2009; Geiger et al., 2010; Geiger et al., 2011; Brandt et al., 2012; Brandt et al., 2015).

In this study, the importance of Ca^2+^ in SL-induced stomatal closure was firstly determined by performing pharmacological studies, which indicate that Ca^2+^ acts as a prominent mediator functions in SL induction of stomatal closure. Through examining a collection of *cpk* mutants, we identified CPK33 (and possibly CPK10) which is implicated in guard cell SL signaling. SL activation of stomatal closure, as well as Ca^2+^-induced stomatal closure, was greatly impaired in *cpk33* mutants. Additionally, CPK33 kinase activity is essential for SL induction of stomatal closure. We further found that H_2_O_2_-induced stomatal closure was moderately impaired in *cpk33* mutants whereas SL-mediated H_2_O_2_ production was maintained in *cpk33* mutants. Taken together, our study uncovers an important player CPK33 that functions as an important mediator in SL signaling in *Arabidopsis* guard cells.

## Materials and Methods

### Plant Growth Conditions and Mutants Isolation

The *Arabidopsis* ecotype Columbia-0 (Col-0) was used as wild-type (WT) plants in this study. The following mutants have been described previously: *max1-1*, *max2-1*, *max2-2*, *max3-9*, and *max4-1* (Stirnberg et al., 2002; Umehara et al., 2008), *d14-5* (Yao et al., 2016), *cpk4-1* and *cpk11-2* (Zhu et al., 2007), *cpk8* (Zou et al., 2015), *cpk10* (Zou et al., 2010), *cpk23* (Ma and Wu, 2007), *cpk33-1*, *cpk33-2*, *35S::CPK33* and *35S::CPK33^K102R^* (Li C.L et al., 2016), *cpk3 cpk5 cpk6 cpk11* (Guzel Deger et al., 2015), and *cpk5 cpk6 cpk11 cpk23* (Wang et al., 2018).

To obtain the double mutant *cpk10 cpk33*, the *cpk10* mutant and *cpk33* mutants (*cpk33-1* and *cpk33-2*) were crossed. Homozygous double mutants were determined by PCRs using a combination of a gene-specific primer and a T-DNA border primer. The primers used were listed in **Table S1**.

Seeds were sterilized and sown for germination in 1/2 Murashige and Skoog (MS) medium supplemented with 0.8% (w/v) agar and 1% sucrose (w/v). Seedlings were transplanted into pots and subsequently kept in a growth chamber with a 16h/8h (light/darkness) regime at 21°C. Fully expanded rosette leaves detached from 4- to 6-week-old healthy plants were harvested for immediate use.

### Stomatal Aperture Bioassay

Stomatal apertures were measured as described previously (Lv et al., 2018; Zhang et al., 2019). In brief, epidermal strips of fully expanded leaves were incubated in the MES-KCl buffer (10 mM MES-KOH/50 mM KCl, pH 6.15) to promote stomatal opening following treatments as described in each experiment. Finally, stomatal apertures on the abaxial epidermis were measured and presented as mean ± SE of three replicates.

### Chemicals

The preparation of synthetic SL analog GR24 (Chiralix Nijmegen, the Netherlands) and 2′,7′-dichlorofluorescein diacetate (H_2_DCF-DA; Biotium Hayward, USA) were performed as described previously (Lv et al., 2018; Zhang et al., 2019). The ethylene glycol-bis(β-aminoethyl ether)-N,N,N′,N′-tetra acetic acid (EGTA; Sigma, USA), lanthanum chloride (LaCl_3_; Solarbio, China), aluminum chloride (AlCl_3_, Solarbio, China), trifluoperazine (TFP), and trifluoperazine dihydrochloride (TFP; Santa Cruz, USA) were dissolved appropriately according to the supplier information and prepared stock solutions for further use. Other chemicals used in this study, including H_2_O_2_, 2-(N-morpholino) ethanesulphonic acid (MES), and calcium chloride (CaCl_2_), were purchased from Sigma-Aldrich. All chemicals used are of the highest analytical grade.

### H_2_O_2_ Content Detection in Guard Cells

H_2_DCF-DA was utilized to determine H_2_O_2_ content in stomata. The measurement of H_2_O_2_ content was performed according to Lv et al. (2018) and Zhang et al. (2019). Briefly, epidermal strips with open stomata were incubated in the Tris-KCl solution containing 50 µM H_2_DCF-DA for 10 min of darkness at 25°C in the absence or presence of GR24. Epidermal strips were washed three times with Tris-KCl buffer to remove excess dye under darkness. The fluorescence in stomata was visualized using a TCS SP2 confocal laser scanning microscope (Leica Lasertechnik GmbH, Germany). The fluorescence intensity representing the endogenous H_2_O_2_ content was determined using ImageJ software. Fluorescence intensities were normalized to those of controls. The data for fluorescence intensities represent the mean ± SE of three replicates.

### Statistical Analyses

Statistical analyses were performed using a one-way ANOVA to discriminate significant differences followed by the least significant difference test.

## Results

### Ca^2+^ Is Required for SL-Induced Stomatal Closure in *Arabidopsis*

**Figure 1:**
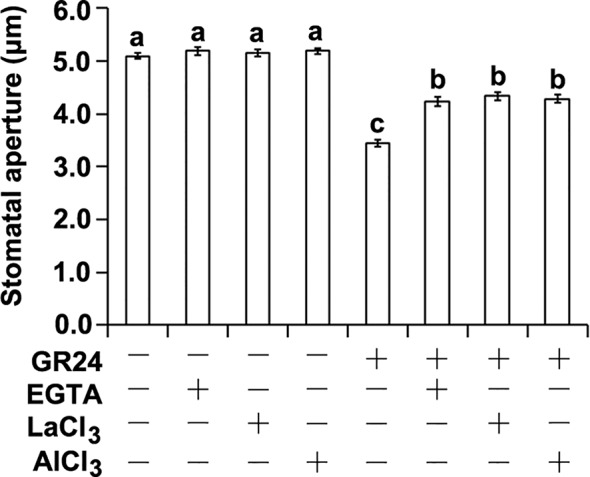
The effects of Ca^2+^ chelator and channel inhibitors on strigolactone-induced stomatal closure. Leaf epidermal peels of WT plants with open stomata were exposed to either the MES-KCl buffer with (+) or without (–) 1 μM GR24, 2 mM EGTA, 1 mM LaCl_3_, or 1 μM AlCl_3_. The stomatal apertures were measured for different treatments. Data are means ± SE of three independent experiments Bars with different letters represent statistically significant differences.

Calcium is known to act as a key secondary messenger in mediating stomatal closure (Blatt, 2000; Bowler and Fluhr, 2000; Pei et al., 2000; Kim et al., 2010; Murata et al., 2015; Ray, 2017). We therefore took a pharmacological approach to determine whether Ca^2+^ is required for SL-triggered stomatal closure. To this end, we examined the effects of ethylene glycol-bis(β-aminoethyl ether)-N,N,N′,N′-tetra acetic acid (EGTA; a Ca^2+^ chelator), and LaCl_3_ and AlCl_3_ (Ca^2+^ channel blockers) on SL-induced stomatal closure (Schwartz, 1985; Zhao et al., 2007; Li Y et al., 2016). As expected, the synthetic SL analog GR24 could significantly induce stomatal closure (**Figure 1**; Lv et al., 2018). However, SL-induced stomatal closure was inhibited by EGTA, LaCl_3_, and AlCl_3_, respectively (**Figure 1**). These results indicate that Ca^2+^ and Ca^2+^ channels are involved in SL-induced stomatal closure.

It was reported that exogenous Ca^2+^ could promote stomatal closure (Blatt, 2000; Ray, 2017). Since we have shown that Ca^2+^ plays a crucial role in SL-induced stomatal closure, we further examined the stomatal response of SL-related mutants in response to Ca^2+^. We found that addition of Ca^2+^ (CaCl_2_) induced a similar stomatal closure in SL-related mutants as in WT plants, whereas SL-signaling mutants *max2* and *d14* were insensitive to GR24 as reported previously (**Figure S1**; Lv et al., 2018). This result indicates that Ca^2+^ might act as a signaling molecule downstream of SL-signaling and SL-biosynthetic genes.

### SL- and Ca^2+^-Induced Stomatal Closure Were Impaired in the *Cpk33* Mutant

Given that Ca^2+^ is essential for SL-induced stomatal closure (**Figure 1**), we sought to identify the specific Ca^2+^ sensor(s) that is involved in guard-cell SL signaling. CPKs, as Ca^2+^ sensors, are important in the regulation of ABA-mediated and Ca^2+^-mediated stomatal closure (Kim et al., 2010; Boudsocq and Sheen, 2013; Schulz et al., 2013; Simeunovic et al., 2016). To determine whether any CPK is required for SL signaling in guard cells, we firstly examined SL-induced stomatal closure in the absence and presence of CPK inhibitors TFP and staurosporine (ST) (Li et al., 1998). TFP and ST significantly inhibited GR24-induced stomatal closure (**Figure S2**), suggesting that CPK(s) is truly indispensable for SL-induced stomatal closure.

**Figure 2:**
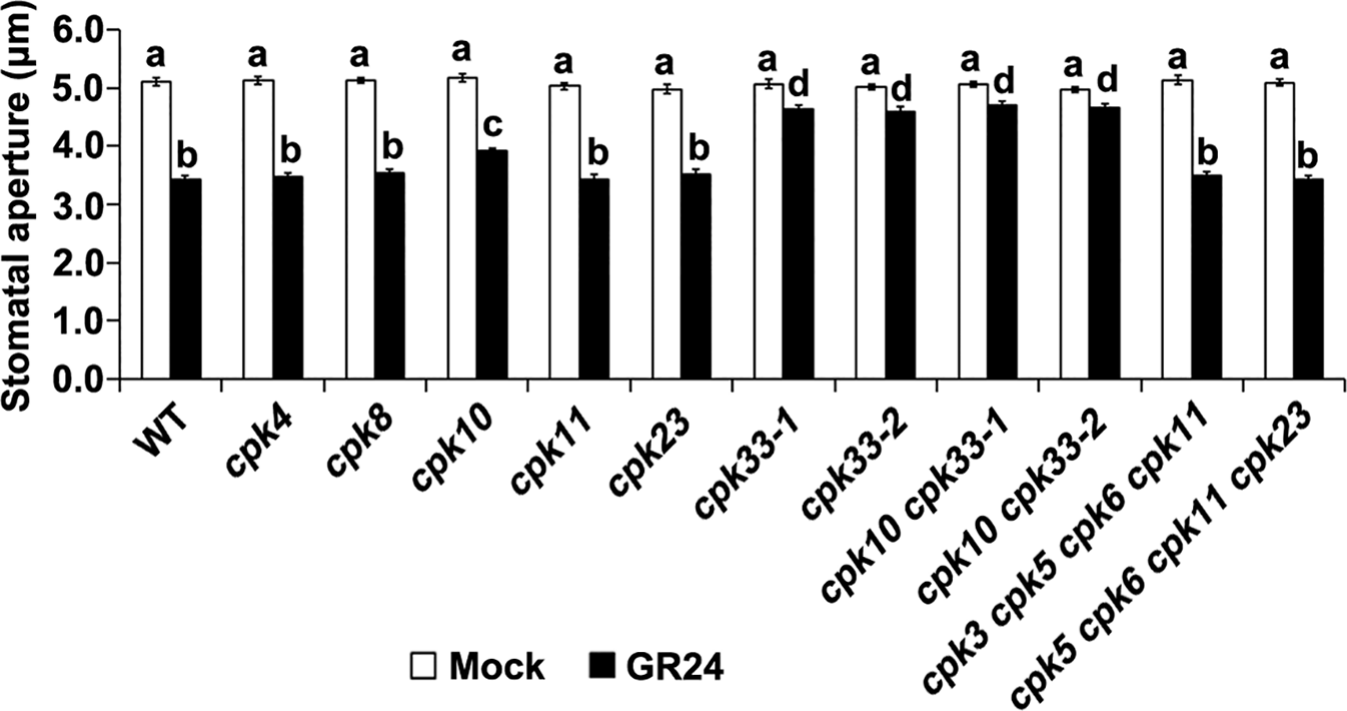
Strigolactone-stimulated stomatal closure is mediated by CPK33. Leaf epidermal peels of WT plants and a collection of *cpk* mutants were exposed to the MES-KCl buffer in the absence and presence of 1 μM GR24. Stomatal apertures were measured and presented as means ± SE of three independent experiments. Bars with different letters represent statistically significant differences.

Furthermore, to identify CPK(s) that might be involved in SL signaling in guard cells, the effect of GR24 on stomatal aperture was examined in a collection of *cpk* mutants, including *cpk4*, *cpk8*, *cpk10*, *cpk11*, *cpk23*, *cpk33-1*, *cpk33-2*, *cpk3 cpk5 cpk6 cpk11*, and *cpk5 cpk6 cpk11 cpk23* (**Figure 2**). GR24 significantly induced stomatal closure in most of examined *cpk* mutants. Conversely, SL-induced stomatal closure is greatly impaired in *cpk33-1* and *cpk33-2* mutants, and marginally impaired in *cpk10* mutants (**Figure 2**). To overcome the potential redundant function between CPK10 and CPK33, we generated double mutants *cpk10 cpk33*-*1* and *cpk10 cpk33*-*2*. The double mutants *cpk10 cpk33* exhibited insensitivity to SL-induced stomatal closure to a similar extent to that of the *cpk33* mutants (**Figure 2**), suggesting that CPK10 exerted slight effect on SL-induced stomatal closure. Nevertheless, we found that CPK33 is predominantly involved in guard cell SL signaling, and we thus concentrated on CPK33 in subsequent studies.

**Figure 3:**
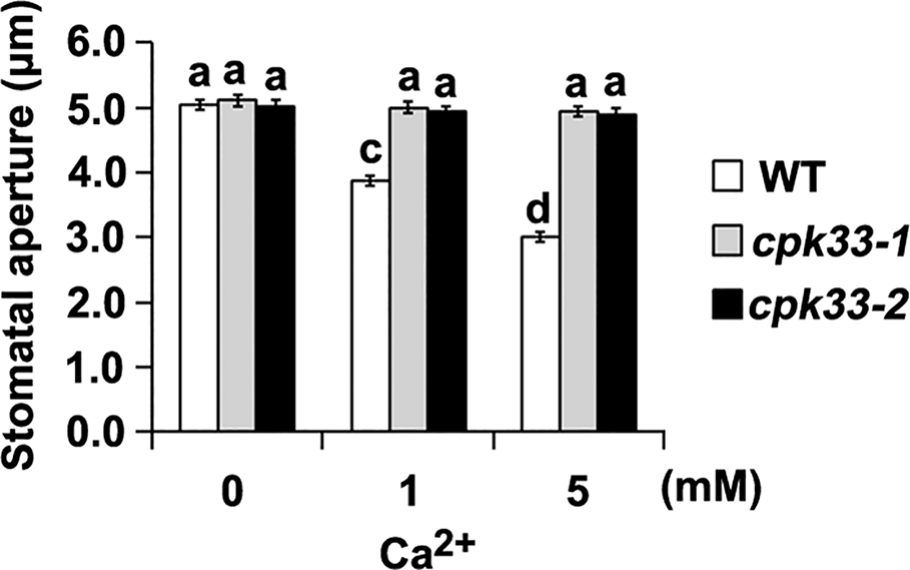
Ca^2+^-induced stomatal closure is significantly impaired in *cpk33* mutants. Leaves of WT plants, *cpk33-1*, and *cpk33-2* mutants with open stomata were exposed to the MES-KCl buffer containing different concentrations of CaCl_2_. Stomatal apertures were subsequently measured and presented as means ± SE of three independent experiments. Bars with different letters represent statistically significant differences.

Because SL promotion of stomatal closure depends on Ca^2+^, we hypothesized that disruption of *CPK33* would impair Ca^2+^ signaling transduction. To test this hypothesis, we investigated the stomatal response following exogenous Ca^2+^ application in *cpk33*-*1* and *cpk33*-*2* mutants. Exogenous Ca^2+^ stimulated stomatal closure in WT plants, but not in *cpk33*-*1* and *cpk33*-*2* mutants (**Figure 3**). Taken together, our results indicate that CPK33 acts as an important Ca^2+^ sensor that is involved in guard-cell SL signaling.

### CPK33 Kinase Activity Is Essential for SL-Induced Stomatal Closure

**Figure 4:**
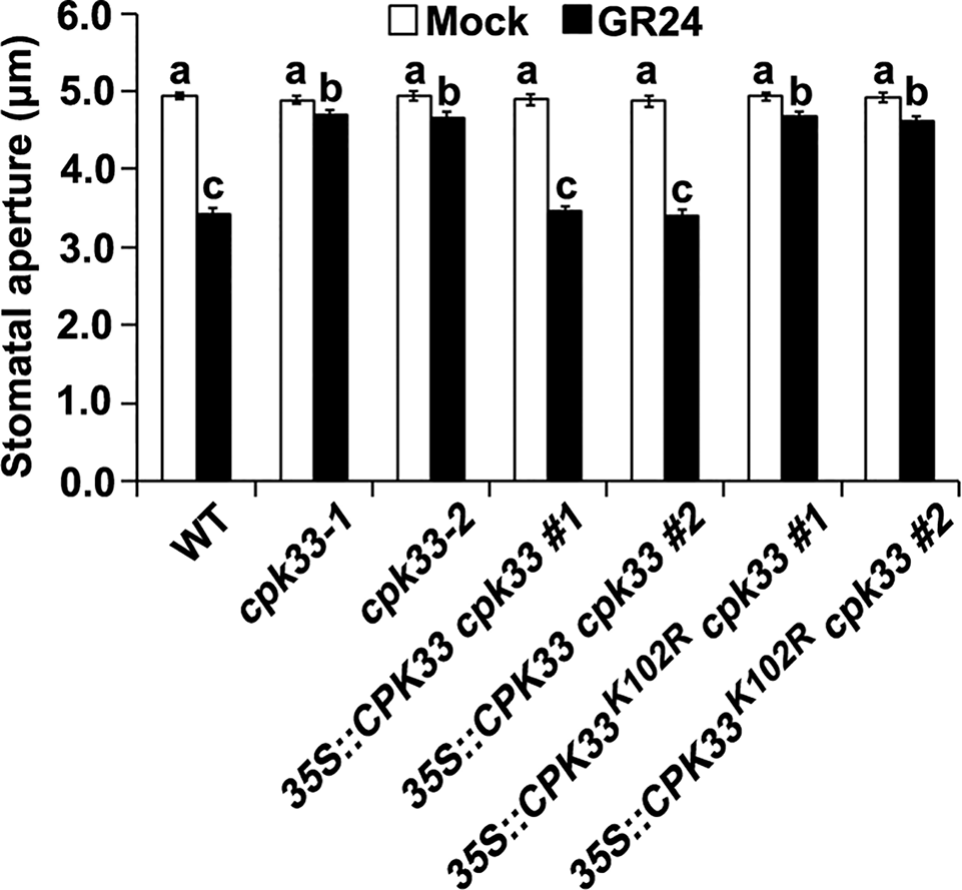
CPK33 kinase activity is essential for strigolactone-induced stomatal closure. Leaves of WT, *cpk33-1*, *cpk33-2*, *35::CPK33 cpk33 #1*, *35::CPK33 cpk33 #2*, *35::CPK33^K102R^ cpk33 #1*, and *35::CPK33^K102R^ cpk33 #2* plants with open stomata were exposed to the MES-KCl buffer in the absence or presence of 1 µM GR24. Stomatal apertures were measured and presented as means ± SE of three independent experiments. Bars with different letters represent statistically significant differences.

Previous studies have shown that the kinase activity of CPK33 is essential for SLAC1 channels and GORK channels activities and ABA-induced stomatal movement (Li C.L et al., 2016; Corratgé-Faillie et al., 2017). To investigate whether the *in vivo* kinase activity of CPK33 is also required for SL-induced stomatal closure, we performed stomatal bioassay analysis upon GR24 treatment using *cpk33-1* and *cpk33-2* mutants, two independent *cpk33-1* complementation lines (*35::CPK33 cpk33* #1 and #2), and two independent lines expressing a kinase-inactive CPK33^K102R^ construct in *cpk33-1* (*35::CPK33^K102R^ cpk33* #1 and #2). Two *35::CPK33 cpk33* lines exhibited GR24-sensitive phenotype similar to that of WT plants, while we found that *35::CPK33^K102R^ cpk33* plants, as well as *cpk33*-*1* and *cpk33*-*2* mutants, failed to close stomata in response to GR24 (**Figure 4**). Thus, this result suggests that CPK33 kinase activity is essential for stomatal closure induced by SLs.

### Effects of the *cpk33* Mutation on SL-Induced H_2_O_2_ Production and H_2_O_2_-Mediated Stomatal Closure in Guard Cells

**Figure 5:**
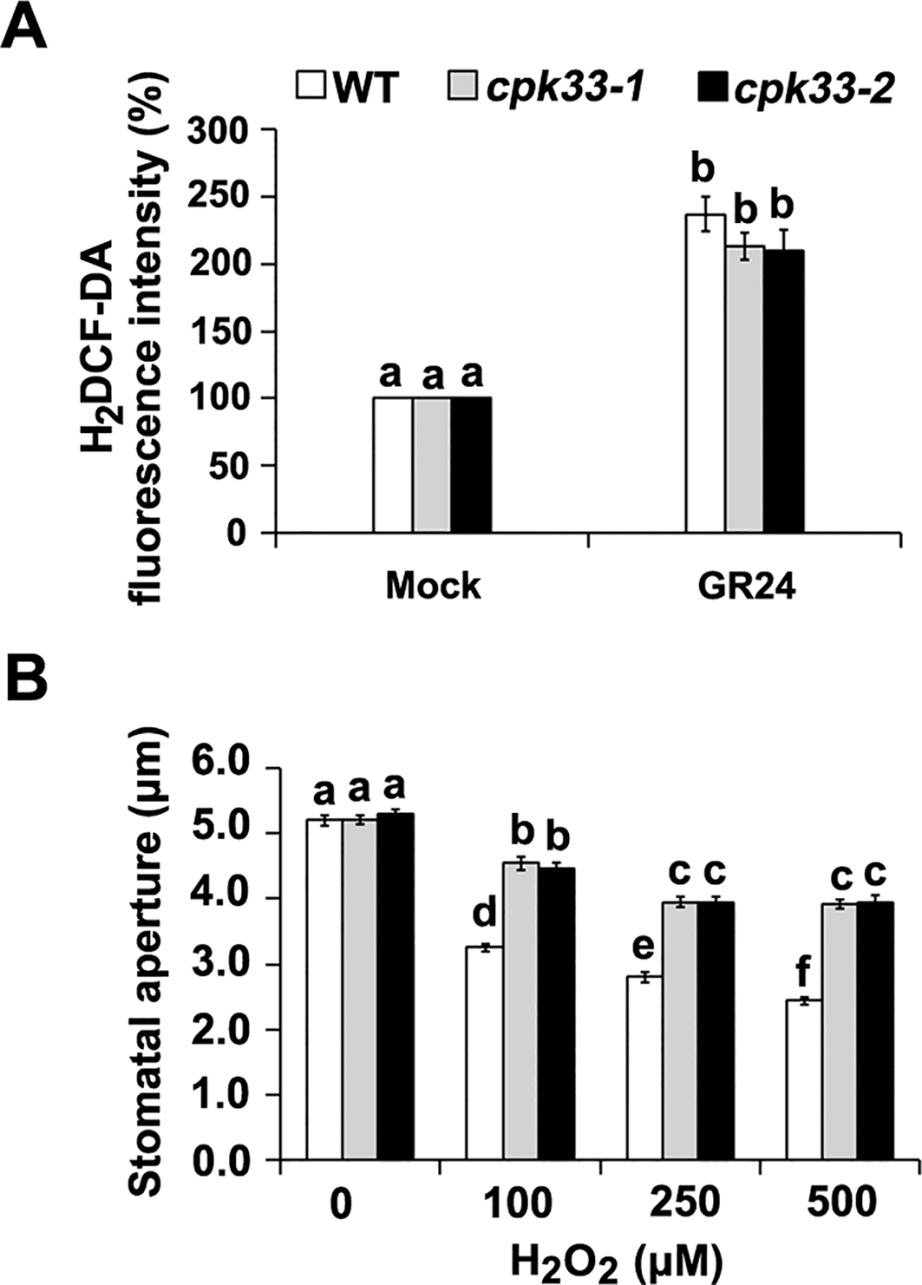
The role of CPK33 in strigolactone-induced H_2_O_2_ production and H_2_O_2_-mediated stomatal closure in guard cells. **(A)** Effects of GR24 on H_2_O_2_ production in guard cells of WT plants, *cpk33-1* and *cpk33-2* mutants. Fluorescence intensities were normalized to those of controls that were taken as 100% for the indicated experiments. **(B)** Epidermal strips of WT plants with open stomata were exposed to the MES-KCl buffer containing different concentrations of H_2_O_2_. The stomatal apertures were measured and presented as means ± SE of three independent experiments. Bars with different letters represent statistically significant differences.

It was reported previously that SLs stimulate H_2_O_2_ accumulation, and the resultant H_2_O_2_ acts as an early signal component in the induction of stomatal closure triggered by SLs (Lv et al., 2018). To investigate the genetic relationship between H_2_O_2_ and CPK33 in guard cell SL signaling, we examined the SL effect on H_2_O_2_ production in *cpk33* mutants using the H_2_O_2_ fluorescent probe H_2_DCF-DA. In line with previous data, GR24 stimulated H_2_O_2_ production in WT plants. Similarly, GR24 also stimulated H_2_O_2_ production in *cpk33* mutants (**Figure 5A**), suggesting that CPK33 disruption did not affect SL-induced H_2_O_2_ production.

To further investigate the position of H_2_O_2_ production and CPK33 in guard cell SL signaling pathway, we next examined the stomatal aperture upon exogenous H_2_O_2_ in *cpk33* mutants. Exogenous application of H_2_O_2_ significantly stimulated stomatal closure in WT plants, but H_2_O_2_-induced stomatal closure is partially impaired in *cpk33* mutants in comparison with WT plants (**Figure 5B**). Altogether, our results indicate that CPK33 possibly functions downstream of H_2_O_2_ production in guard-cell SL signaling. It appears that, as a paradigm, H_2_O_2_ activates CPK(s) that function as signal mediator(s) in guard cells. However, it remains to be explored whether and how CPK33 is activated by SL-induced H_2_O_2_

## Discussion

It has been shown that SLs were implicated in various developmental processes of plants and mediate their responses to environmental stress (Al-Babili and Bouwmeester, 2015; Marzec, 2016; Cardinale et al., 2018; Mostofa et al., 2018). Recently we found that SLs function as common regulators to induce stomatal closure (Lv et al., 2018; Zhang et al., 2018). We further elucidated that SL-induced stomatal closure is accomplished through enhancing production of H_2_O_2_ and nitric oxide, eventually promoting plant resilience to environmental stress (Lv et al., 2018; Zhang et al., 2018). However, no significant effect on stomatal aperture was observed when spraying GR24 onto intact plants, although SL-related mutants were genetically confirmed to display higher stomatal conductance (Kalliola et al., 2019). The discrepancy in SL-induced stomatal closure may be due to the different materials used, intact plants and epidermal strips, which possibly results in different efficacy of GR24 such as permeability problem. It has been reported that Ca^2+^ and its sensors CPKs are crucial for stomatal closing (Blatt, 2000; Bowler and Fluhr, 2000; Pei et al., 2000; Kim et al., 2010; Murata et al., 2015; Ray, 2017). OST1, a Ca^2+^-independent kinase, was found to be dispensable for SL-induced stomatal closure (Lv et al., 2018). Based on these observations, we thus hypothesize that Ca^2+^ as well as its transducers CPK(s) is implicated in SL-triggered stomatal closure.

In this study we found that Ca^2+^ chelator EGTA, and Ca^2+^ channel blockers LaCl_3_ and AlCl_3_ significantly suppressed SL-induced stomatal closure (**Figure 1**), suggesting the requirement of extracellular free Ca^2+^ and Ca^2+^ channels in the modulation of SL-induced stomatal closure. Our pharmacological and further genetic analyses suggest that SL requires cytosolic Ca^2+^ signals to promote stomatal closure (**Figures 1** and **3**). It is possible that SLs stimulate either Ca^2+^ sensitivity priming or cytosolic Ca^2+^ elevation to perturb Ca^2+^ signals, thereby resulting in stomatal closure. It is thus demanded to detect the alteration of Ca^2+^ signals through Ca^2+^ detection fluorescence dyes and/or different Ca^2+^ biosensors in the future analyses.

It has long been known that CPKs, acting as Ca^2+^ sensors, are important mediators of Ca^2+^-dependent stomatal closure and ion channel activation (Kim et al., 2010; Boudsocq and Sheen, 2013; Schulz et al., 2013; Simeunovic et al., 2016). We identified a potential Ca^2+^ transducer CPK33 acting as an intermediate component downstream of H_2_O_2_ in guard cell SL signaling pathway. SL activation of stomatal closure, as well as Ca^2+^-induced stomatal closure, were impaired in *cpk33* mutants (**Figures 2** and **3**). Interestingly, CPK10 disruption slightly impaired SL-induced stomatal closure (**Figure 2**). The double mutant *cpk10 cpk33* exhibited insensitivity to SL-induced stomatal closure to a similar extent to that of the *cpk33* mutant (**Figure 2**), suggesting a prime role of CPK33 and a differential contribution of CPK10 and CPK33 in SL-induced stomatal closure. It was found previously that CPK10 was involved in plant responses to drought stress *via* modulation of ABA- and Ca^2+^-mediated stomatal closure (Zou et al., 2010). Considering the complexity of crosstalk of distinct signaling pathways, it is plausible that CPK10 might indirectly impact on the SL activation of stomatal closure. Alternatively, given that there are 34 CPKs in *Arabidopsis* (Boudsocq and Sheen, 2013), a yet unidentified CPK, rather than CPK33, may function redundant with CPK10 in modulation of SL-induced stomatal closure. Nevertheless, the role of CPK10 (possible with another redundant CPK) in SL promotion of stomatal closure needs to be investigated.

Intriguingly, guard cell outward potassium channel GORK is specifically stimulated by CPK33 to active outward potassium ion currents, showing that, unlike its negative regulation of anion channels (Li C.L et al., 2016), CPK33 positively modulates the GORK channel activity to promote stomatal closure (Corratgé-Faillie et al., 2017). Consequently, *cpk33* mutants were delayed in stomatal closure under normal conditions (Corratgé-Faillie et al., 2017). In this regard, it is possible that SLs could exploit CPK33-activated GORK channels to promote stomatal closure. It thus remains to be investigated whether SLs could stimulate CPK33-activated GORK channel activity. CPK33 was reported as a negative regulator in ABA-modulated stomatal closure (Li C.L et al., 2016). SL activation of stomatal closure, however, were inhibited in *cpk33* mutants (**Figure 2**), indicating that CPK33 positively functions in SL-induced stomatal closure. Taken together, our results and previous reports suggest that the CPK-dependent Ca^2+^ recognition conveying by CPK33 could be essential for SL signaling, as well as ABA signaling in guard cells. Nevertheless, the role of opposite effect of CPK33 on anion and potassium channels, as well as the discrepant role of CPK33 in guard cell ABA and SL signaling, is required to be further studied.

**Figure 6:**
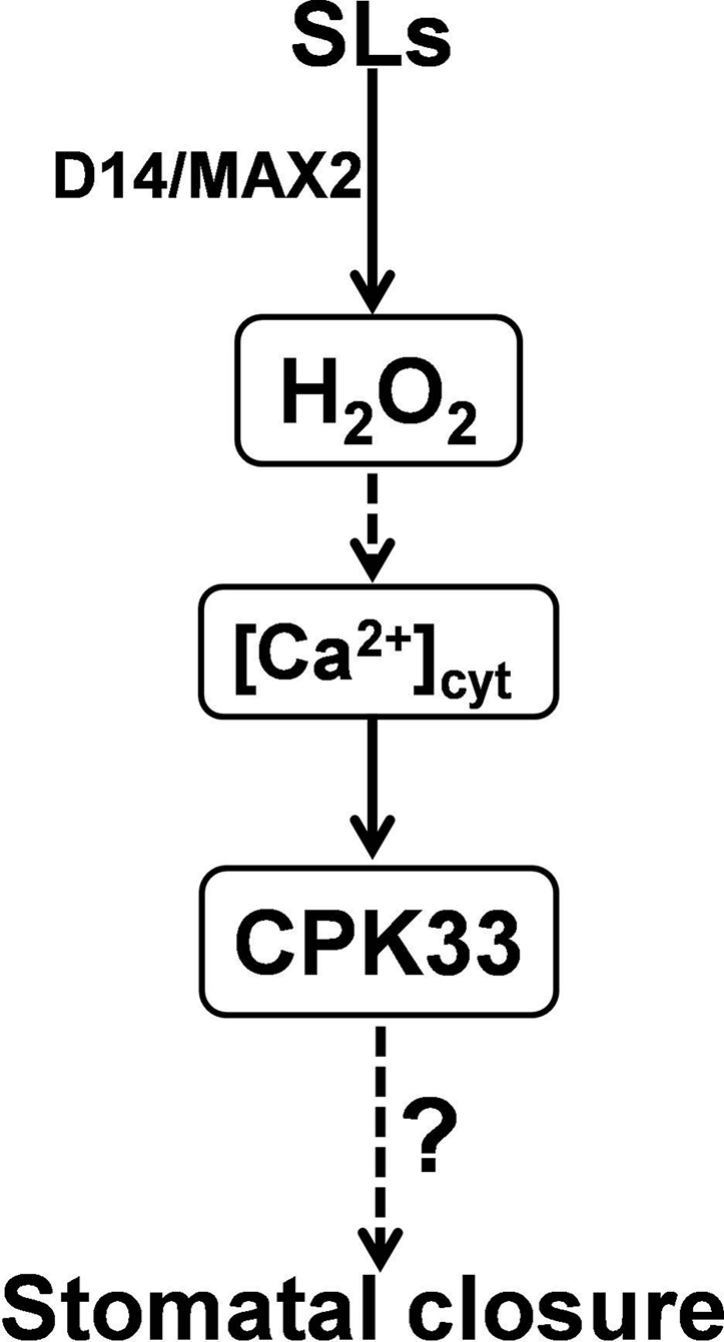
A working model for CPK33-mediated strigolactone (SL) signaling in guard cells. Following perception by D14 and MAX2, SLs stimulate the production of H_2_O_2_ that possibly activates the Ca^2+^ transducer CPK33 which likely modulates anion and potassium channels to promote stomatal closure. The question mark stands for an unknown molecule(s) that is downstream of CPK33 in SL-mediated stomatal closure. It remains to be explored whether and how SLs and/or SL-induced H_2_O_2_ could promote cytosolic Ca^2+^ which is presumably sensed by CPK33 in SL-mediated guard cell signaling.

Integrated previous studies with our present genetic and physiological analyses, we propose that CPK33, as a Ca^2+^ transducer, acts downstream of H_2_O_2_ and Ca^2+^ (**Figure 6**). In guard cells, following perception by D14 and MAX2, SLs stimulate the production of H_2_O_2_ that possibly activates CPK33, which likely modulates anion and potassium channels to promote stomatal closure (Li C.L et al., 2016; Corratgé-Faillie et al., 2017; **Figure 6**). Indeed, it has been reported that CPK33 positively stimulates the GORK channels while negatively regulates SLAC1 channels to promote stomatal closure (Li C.L et al., 2016; Corratgé-Faillie et al., 2017). However, it remains to be examined whether and how CPK33 is activated by SL-induced H_2_O_2_. It has been reported that H_2_O_2_-stimulated cytosolic Ca^2+^ elevation is crucial for stomatal closure (Pei et al., 2000). CPK33 was confirmed to be a typical Ca^2+^-dependent kinase (Corratgé-Faillie et al., 2017), indicating that CPK33 activity requires cytosolic Ca^2+^ increase. Therefore, it will be intriguing to investigate whether SLs and/or SL-induced H_2_O_2_ are able to promote cytosolic Ca^2+^ which presumably is sensed by CPK33 in SL-mediated guard cell signaling. The finding that the stomata of *cpk33* mutants remain considerable response to H_2_O_2_ indicates that other factor(s) might also required for SL-triggered stomatal closure. In the meanwhile, our results emphasizes the essential role of H_2_O_2_ serving as a hub in the complicated hormone-mediated stomatal closure. Altogether, our study reinforces the understanding of the molecular mechanism by which SLs induce stomatal closure and provides new insights to improve stress acclimatization of plants.

## Data Availability Statement

The datasets generated for this study are available on request to the corresponding author.

## Author Contributions

YZ and GW designed the experiments. XW, SL, XH, XG, XS, JK, ZL, and YZ performed the experiments. BC assisted in microscope. WZ contributed materials. YZ, CL, and GW wrote the manuscript. All authors reviewed the manuscript.

## Funding

The study is supported by the National Natural Science Foundation of China (31701294 and 31801210), the Fundamental Research Funds for Central Universities (GK201702016), the SNNU Laboratory Technology Research Funds (SYJS201739), the Start-up Foundation of Hubei University of Medicine (2016QDJZR14), and the Natural Science Foundation of Hubei Provincial Department of Education (Q20182104).

## Conflict of Interest

The authors declare that the research was conducted in the absence of any commercial or financial relationships that could be construed as a potential conflict of interest.
