# Supplementary figures and images for: The Calcium-Dependent Protein Kinase CPK33 Mediates Strigolactone-Induced Stomatal Closure in Arabidopsis thaliana
Source: Front Plant Sci. 2019 Dec 17;10:1630. doi: 10.3389/fpls.2019.01630 (PMC6928132; doi:10.3389/fpls.2019.01630)

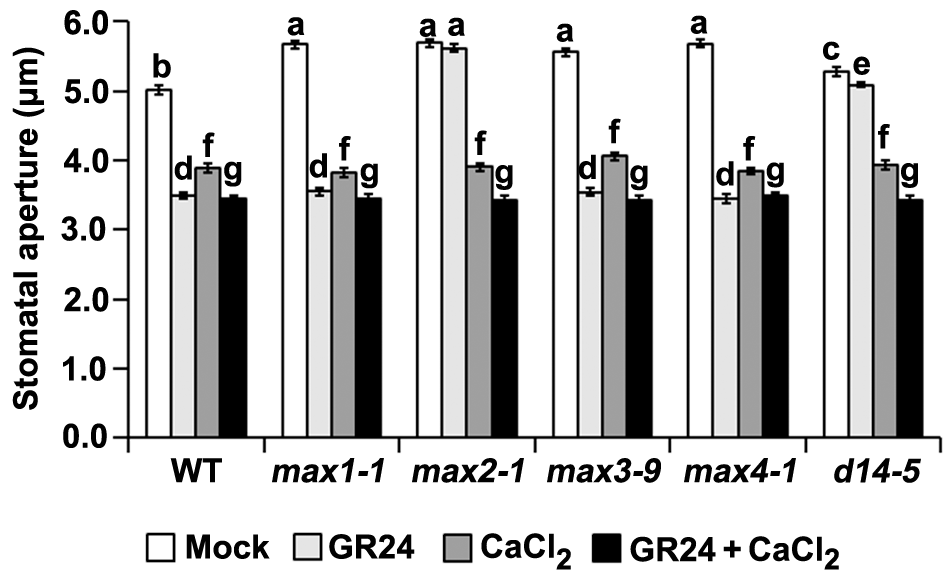

Supplement: Supplementary file 1 [file Image_1.tif]

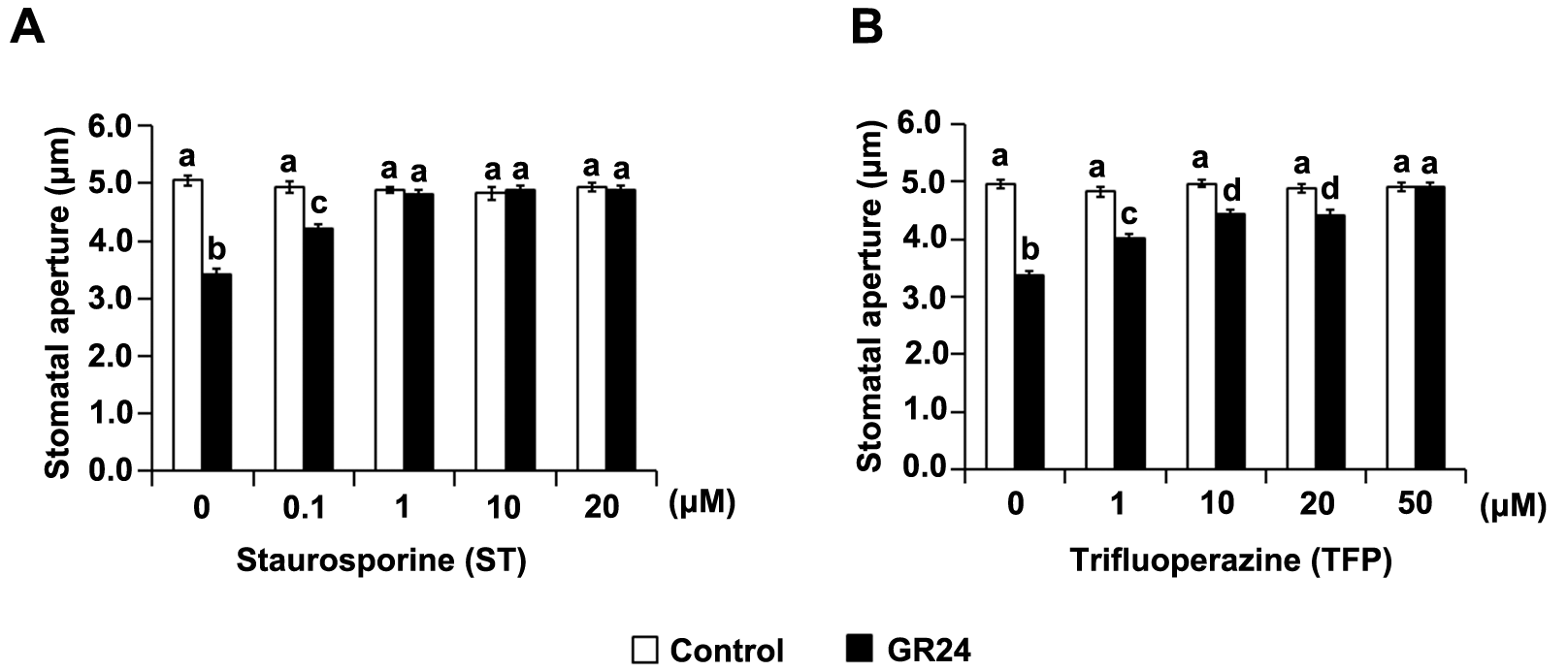

Supplement: Supplementary file 2 [file Image_2.tif]
