# Supplementary material for: The Calcium-Dependent Protein Kinase CPK33 Mediates Strigolactone-Induced Stomatal Closure in Arabidopsis thaliana
Source: Front Plant Sci. 2019 Dec 17;10:1630. doi: 10.3389/fpls.2019.01630 (PMC6928132; doi:10.3389/fpls.2019.01630)
Supplement: Supplementary file 3 [file Table_1.docx]

**Table S1. List of primers used in this study for genotyping.**

| **Primer names** | **Sequences (5' to 3')** |
| --- | --- |
| Salk LB primer | TGGTTCACGTAGTGGGCCATCG |
| CPK10-LP | ATCCTGATCCGACTAAGCG |
| CPK10-RP | CCAACAATCCGACTCAGAA |
| CPK33-1- LP | AGTGAAACAGCTCATGGATGC |
| CPK33-1-RP | ATGACACACAGGTTACTCGGC |
| CPK33-2-LP | TGTTGTGGATTCCCACTTCTC |
| CPK33-2-RP | TGGAAGGTGAAATCGACTTTG |
